# Samul-Tang Regulates Cell Cycle and Migration of Vascular Smooth Muscle Cells against TNF-*α* Stimulation

**DOI:** 10.1155/2018/1024974

**Published:** 2018-06-25

**Authors:** Eun Sik Choi, Jung Joo Yoon, Byung Hyuk Han, Da Hye Jeong, Hye Yoom Kim, You Mee Ahn, So Young Eun, Yun Jung Lee, Dae Gill Kang, Ho Sub Lee

**Affiliations:** ^1^College of Oriental Medicine and Professional Graduate School of Oriental Medicine, Wonkwang University, 460 Iksan-daero, Iksan-si, Jeollabuk-do 54538, Republic of Korea; ^2^Hanbang Cardio-Renal Syndrome Research Center, Wonkwang University, 460 Iksan-daero, Iksan-si, Jeollabuk-do 54538, Republic of Korea

## Abstract

Samul-Tang (SMT), consisting of four medicinal herbs, is a well-known herbal prescription treating hematological disorders related symptoms. Our previous study demonstrated that SMT attenuated inflammation of vascular endothelial cells. In condition of retained vascular dysfunction, vascular inflammation is initiated and results in activation of smooth muscle cells (SMCs). Activated SMCs lose control of cell cycle regulation and migrate into intima, resulting in formation of atheroma. Here, we further investigated whether SMT suppresses proliferation and migration of SMCs. SMT showed antiproliferative effects on SMCs by suppressing [^3^H]-thymidine incorporation against TNF-*α* stimulation. Underlying mechanisms of antiproliferative effects were found to be resulting from cell cycle regulation. SMT downregulated expression of cyclin D1-CDK4 and cyclin E-CDK2 complexes and upregulated p21^waf1/cip1^ and p27^kip1^. SMT also suppressed migration of SMCs against TNF-*α* stimulation. This is thought to have resulted from suppressing MMP2 and MMP9 expressions and ROS production. In summary, SMT attenuates abnormal migration of vascular smooth muscle cells via regulating cell cycle and suppressing MMPs expression and ROS production. Our study suggests that SMT, a traditionally used herbal formula, protects vascular smooth muscle cells and might be used as an antiatherosclerotic drug.

## 1. Introduction

The vascular smooth muscle cells (SMCs) are located in the walls of blood vessels and regulate contraction or relaxation of blood vessel. Besides, SMCs are also involved in developing cardiovascular complication such as atherosclerosis resulting from chronic vascular inflammation or abnormal proliferation and migration of SMCs [1].

In pathogenesis of atherosclerosis, proliferation and migration of SMCs are crucial [2] and regulating cell cycle of SMCs could be a novel therapeutic strategy. Basically, progression of cell cycle is positively regulated by cyclin-cyclin-dependent kinase (CDK) complexes and negatively regulated by CDK inhibitors. In G1 phase, cyclin D1 drives the formation of active CDK4 and cyclin E activates CDK2. Consequently, cyclin D1-CDK4 and cyclin E-CDK2 complexes drive cells to exit G1 phase and initiate DNA replication to enter into S phase [3]. In contrast, CDK inhibitors such as p21^waf1/cip1^ and p27^kip1^ stabilize cyclin-CDK complexes and negatively regulate cell cycle [4].

Matrix metalloproteinases (MMPs) are enzymes involved in degradation and remodeling of extracellular matrix (ECM). Particularly, MMP2 (72 kDa) and MMP9 (92 kDa), which are also called gelatinase, play important role in breaking down structure of ECM [5] as well as regulating proliferation and migration of SMCs [6]. Excessive breakdown of ECM might lead to plaque rupture and incidence of myocardial infarction could be increased [7]. Oxidative stress mediated by reactive oxygen species (ROS) also prompts vascular inflammation developing atherosclerosis and also activates cell cycle circulation and MMPs [8].

Samul-Tang (SMT, Si-Wu-Tang in Chinese and Four-Agent-Decoction in English) is a herbal prescription consisting of four medicinal herbs: Angelicae Gigantis Radix (*Angelica gigas *Nakai, root), Cnidii Rhizoma (*Ligusticum officinale* Makino, rhizome), Rehmanniae Radix Preparata (*Rehmannia glutinosa* Gaertn. DC., rhizome, steamed and dried), and Paeoniae Radix (*Paeonia lactiflora* Pall., root).* Dongui Bogam (Treasured Mirror of Eastern Medicine)* and other several formularies contain medical information about SMT. Traditional use of SMT is treating hematological disorders related symptoms such as anemia [9], irregular menstruation [10, 11], or postpartum weakness defined as blood deficiency and blood stasis in traditional Korean medicine. Recent pharmacological studies suggest that SMT exerts hematopoietic [12], anti-inflammatory [13], and antidermatitis [14] effects. Furthermore, our previous study demonstrated that SMT exerts vascular protective effects in human umbilical vein endothelial cells (HUVECs), which are pathologically intimate with SMCs [15]. Here, we further investigated effects and mechanisms of SMT on tumor necrosis factor-*α* (TNF-*α*) stimulated SMCs proliferation and migration.

## 2. Materials and Methods

### 2.1. Plant Materials of SMT

The four crude herbs forming SMT were purchased from Omniherb (Yeongcheon, Korea) in February 2008. The origin of each herbal medicine was taxonomically identified by Professor Je Hyun Lee, Dongguk University, Gyeongju, Republic of Korea. A voucher specimen (2008-KE25-1~KE25-4) has been deposited at the K-herb Research Center, Korea Institute of Oriental Medicine. SMT extract was prepared as described previously [15].

### 2.2. Chemicals and Reagents

DMEM low glucose, fetal bovine serum, TNF-*α*, cell culture reagents, and CM-H_2_DCFDA were purchased from Invitrogen (San Diego, CA). Primary antibodies, including mouse anti-cyclin D1, rabbit anti-CDK4, mouse anti-cyclin E, rabbit anti-CDK2, rabbit anti-p21, mouse anti-p27, rabbit anti-MMP2, and rabbit anti-MMP9, were purchased from Santa Cruz Biotechnology (CA, USA). Goat anti-rabbit IgG and goat anti-mouse IgG were purchased from Enzo (Farmingdale, USA).

### 2.3. Cell Culture

Human aortic smooth muscle cells (SMCs, C-007-5C) were purchased from Invitrogen (Carlsbad, CA). Cells were cultured with DMEM low glucose containing 5% fetal bovine serum and penicillin-streptomycin and maintained in a humidified incubator containing 5% CO_2_ at 37°C.

### 2.4. [^3^H]-Thymidine Incorporation Assay

Quiescent cells were treated with 10 ng/ml TNF-*α* and SMT, respectively, and 1 *μ*Ci of [^3^H]-thymidine was added (methyl-[^3^H] thymidine, 50 Ci/mmol; Amersham, Oakville, Ontario, Canada). After 24 h of incubation, cells were washed once with 2 ml of ice-cold PBS for 10 min, extracted three times with 2 ml of cold 10% TCA for 5 min each, and solubilized for at least 30 min at room temperature in 0.2 ml of 0.3 N NaOH, 1% SDS. After neutralization with 0.2 ml of 0.3 N HCl, [^3^H]-thymidine activity was measured in a liquid scintillation counter (Beckman LS 7500, Fullerton, CA). Each experiment was conducted either in triplicate or in quadruplicate.

### 2.5. Western Blot Analysis

Cell homogenates were separated on 10% SDS-polyacrylamide gel electrophoresis and transferred to nitrocellulose paper. Blots were then washed with H_2_O, blocked with 5% skimmed milk powder in Tris-Buffered Saline Tween-20 (TBS-T) (10 mM Tris-HCl, pH 7.6, 150 mM NaCl, 0.05% Tween-20) for 1 h, and incubated with the appropriate primary antibody at dilutions recommended by the supplier. Then the membrane was washed, and primary antibodies were detected with secondary antibodies conjugated to horseradish peroxidase, and the bands were visualized with enhanced chemiluminescence (Amersham Bioscience, Buckinghamshire, UK). Protein expression levels were determined by analyzing the signals captured on the nitrocellulose membranes using the ChemiDoc image analyzer (Bio-Rad Laboratories, Hercules, CA).

### 2.6. Cell Migration (Scratch) Assay

The cell migration assay was evaluated by wound healing assay. Briefly, SMCs were plated in 12-well culture plates and cultured in DMEM containing 10% FBS. Scratches were performed with a sterile tip in a 12-well plate containing cells at 80% confluence level. Next, SMCs were pretreated with SMT for 30 min, which was followed by treatment with TNF-*α* at 37°C for 24 h. After incubation, the microscopic photographs of migrated cells were measured by microscopy (Eclipse Ti, Nikon).

### 2.7. Gelatin Zymography

SMCs were pretreated with SMT for 30 min and stimulated with TNF-*α* for 24 h. The supernatant, conditioned medium was collected for zymography. SDS-PAGE for measurement of MMP2 activity was added with 0.1% gelatin in the 10% separated gel. The gel was washed with renaturation buffer (2.5% Triton X-100 in DW) at room temperature for 1 h and then incubated with development buffer (Invitrogen Corporation, Carlsbad, CA) at 37°C overnight. Next, the gel was stained with 0.2% Coomassie brilliant blue R stain reagent at room temperature for 1 h. After washing with destain buffer, the gels were scanned using a ChemiDoc image analyzer (Bio-Rad, USA).

### 2.8. Intracellular ROS Production Assay

The fluorescent probe, (5-(and-6)-chloromethyl-2',7'-dichlorodihydrofluorescein diacetate, acetyl ester) (CM-H_2_DCFDA), was used to determine the intracellular generation of ROS by stimulation of TNF-*α*. Briefly, the confluent SMCs in the 6-well culture plates were pretreated with or without SMT for 30 min. After removing the SMT from the wells, the cells were incubated with 20 *μ*M of CM-H_2_DCFDA for 1 h. The cells were stimulated with TNF-*α*, and the fluorescence intensity was measured at an excitation and emission wavelength of 485 nm and 530 nm, respectively, using a flow cytometry on FACSCalibur (BD, San Diego, CA).

### 2.9. Statistical Analysis

All the experiments were repeated at least three times. The results were expressed as a mean ± SE, and the data were analyzed using one-way ANOVA followed by Student's *t*-test to determine any significant differences. *p* < 0.05 was considered as statistically significant.

## 3. Results

### 3.1. Effects of SMT on TNF-*α* Stimulated SMCs Proliferation

**Figure 1:**
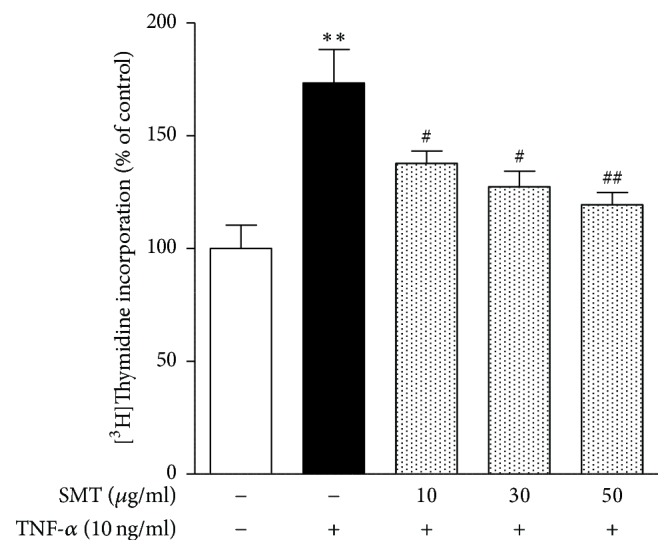
Effects of SMT on TNF-*α* stimulated SMCs proliferation. Cells were treated with TNF-*α* (10 ng/ml) for 24 h in the absence or pretreatment of SMT (10, 30, and 50 *μ*g/ml) for 30 min and incubated with 1 *μ*Ci of [^3^H]-thymidine. [^3^H]-Thymidine incorporation was used as an index of DNA synthesis. Bar represents the mean ± SEM of more than 3 independent experiments. ^*∗∗*^*p* < 0.01 versus untreated control group. ^#^*p* < 0.05 and ^##^*p* < 0.01 versus TNF-*α*-treated group.

To investigate the effects of SMT on proliferation of TNF-*α* stimulated SMCs, [^3^H]-thymidine incorporation assay was performed. [^3^H]-thymidine incorporation is used as an index of DNA synthesis. As shown in Figure 1, stimulation with TNF-*α* for 24 h significantly increased the [^3^H]-thymidine incorporation compared to untreated control group (^*∗∗*^*p* < 0.01), Whereas SMT 10 *μ*g/mL (^#^*p* < 0.05), 30 *μ*g/mL (^#^*p* < 0.05), and 50 *μ*g/mL (^##^*p* < 0.01), respectively, significantly suppressed incorporation of [^3^H]-thymidine against TNF-*α* stimulation in a dose-dependent manner.

### 3.2. Effects of SMT on TNF-*α* Stimulated SMCs Expression of Cell Cycle Regulators

**Figure 2:**
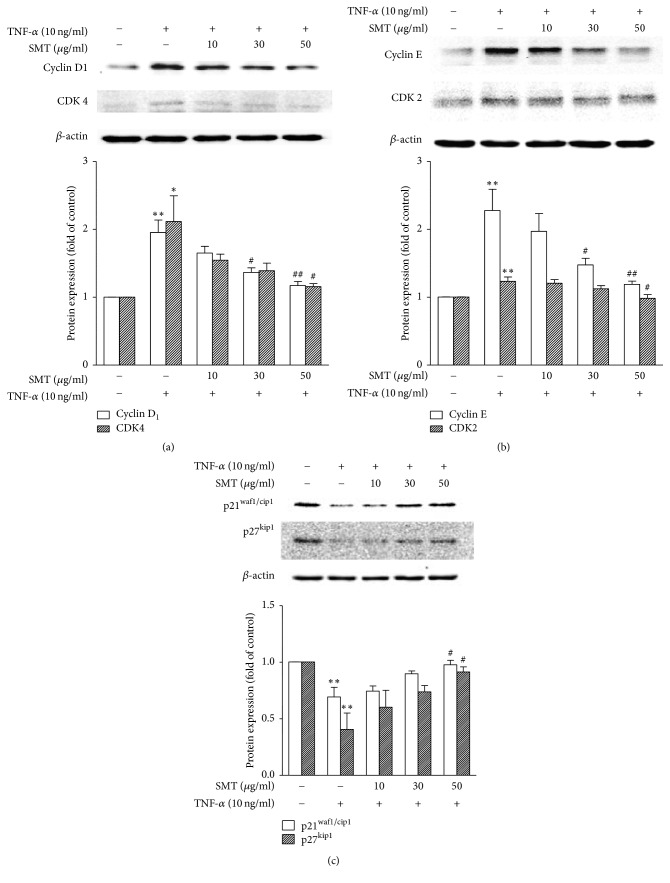
Effect of SMT on TNF-*α* stimulated expression of cell cycle regulators. (a) cyclin D1 and CDK4, (b) cyclin E and CDK2, and (c) p21^waf1/cip1^ and p27^kip1^. Cells were treated with TNF-*α* (10 ng/ml) for 24 h in the absence or pretreatment of SMT (10, 30, and 50 *μ*g/ml) for 30 min. Bar represents the mean ± SEM of 3 independent experiments. ^*∗*^*p* < 0.05 and ^*∗∗*^*p* < 0.01 versus untreated control group. ^#^*p* < 0.05 and ^##^*p* < 0.01 versus TNF-*α*-treated group.

To investigate mechanisms of antiproliferative effects of SMT on SMCs against TNF-*α* stimulation, signaling pathways of cyclin-CDK complexes and CDK inhibitors were assessed by western blot. As shown in Figure 2(a), stimulation with TNF-*α* for 24 h significantly upregulated expression of cyclin D1 (^*∗∗*^*p* < 0.01) and CDK4 (^*∗*^*p* < 0.05) compared to untreated control group, Whereas SMT 30 *μ*g/mL (^#^*p* < 0.05) and 50 *μ*g/mL (^##^*p* < 0.01) significantly suppressed expression of cyclin D1 against TNF-*α* stimulation in a dose-dependent manner. Also, CDK4 expression was suppressed by SMT 50 *μ*g/mL treatment (^#^*p* < 0.05) against TNF-*α* stimulation. As shown in Figure 2(b), stimulation with TNF-*α* for 24 h significantly upregulated expression of cyclin E (^*∗∗*^*p* < 0.01) and CDK2 (^*∗∗*^*p* < 0.01) compared to untreated control group, whereas SMT 30 *μ*g/mL (^#^*p* < 0.05) and 50 *μ*g/mL (^##^*p* < 0.01) significantly suppressed expression of cyclin E against TNF-*α* stimulation in a dose-dependent manner. Also, CDK2 expression was suppressed by SMT 50 *μ*g/mL treatment (^#^*p* < 0.05) against TNF-*α* stimulation. As shown in Figure 2(c), expressions of p21^waf1/cip1^ and p27^kip1^ (CDK inhibitors) were both significantly downregulated by stimulation with TNF-*α* for 24 h (^*∗∗*^*p* < 0.01), whereas SMT 50 *μ*g/mL significantly inhibited downregulation of both p21^waf1/cip1^ and p27^kip1^ against TNF-*α* stimulation.

These results demonstrate that SMT regulates cell cycle of SMCs via inhibiting cyclin and CDK expression and upregulating CDK inhibitors.

### 3.3. Effects of SMT on TNF-*α* Stimulated SMCs Migration

**Figure 3:**
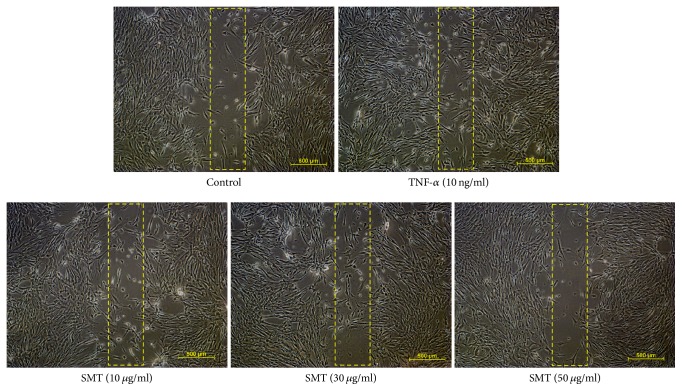
Effect of SMT on TNF-*α* stimulated SMCs migration. Cells were treated with TNF-*α* (10 ng/ml) for 24 h in the absence or pretreatment of SMT (10, 30, and 50 *μ*g/ml) for 30 min. Microscopic photographs of all groups were captured with same magnification (40x) and scale bar indicates 500 *μ*m.

Migration of SMCs is closely related to its proliferation and is a previous process of neointima and plaque formation. Migration assay was performed under examination with microscope and scale bar indicates 500 *μ*m (40x magnification). As shown in Figure 3, TNF-*α* stimulated SMCs showed increased migration compared to untreated control group, whereas SMT-treated SMCs (10–50 *μ*g/mL) showed decreased migration against TNF-*α* stimulation.

### 3.4. Effects of SMT on TNF-*α* Stimulated SMCs Secretion and Expression of MMPs

**Figure 4:**
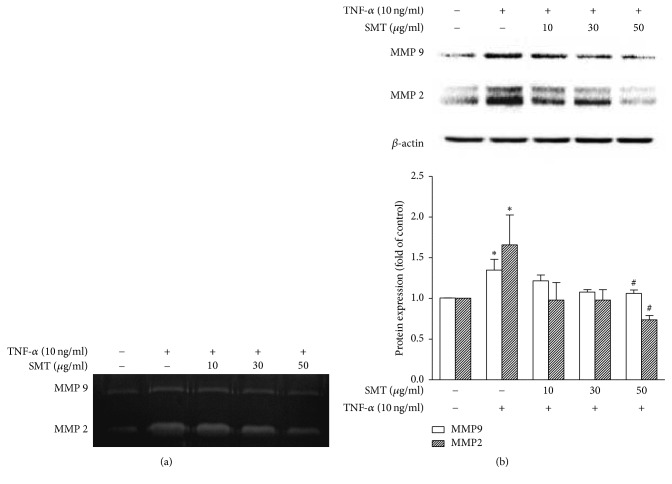
Effect of SMT on TNF-*α* stimulated SMCs secretion and expression of MMPs. Cells were treated with TNF-*α* (10 ng/ml) for 24 h in the absence or pretreatment of SMT (10, 30, and 50 *μ*g/ml) for 30 min. (a) 0.1% gelatin-containing separating gel was used to measure MMPs secretion in zymography. White bands stand for degraded gelatins by MMP9 and MMP2 secreted in cell cultured medium. (b) Protein expression of MMPs. Bar represents the mean ± SEM of 3 independent experiments. ^*∗*^*p* < 0.05 versus untreated control group. ^#^*p* < 0.05 versus TNF-*α*-treated group.

MMP2 and MMP9 are involved in the neointima formation. Gelatin zymography and western blot were performed to measure MMP2 and MMP9 levels. Zymography was performed with cell culture medium incubated with SMCs. As shown in Figure 4(a), zymogram shows that MMP2 and MMP9 secretions of SMCs stimulated with TNF-*α* both increased compared to untreated control group, whereas SMT (10–50 *μ*g/mL) suppressed secretion of MMP2 and MMP9 against TNF-*α* stimulation on SMCs.

As shown in Figure 4(b), protein expression of both MMP2 and MMP9 was significantly upregulated by TNF-*α* stimulation compared to untreated control group (^*∗*^*p* < 0.05), whereas SMT 50 *μ*g/mL significantly suppressed protein expression of both MMP2 and MMP9 against TNF-*α* stimulation (^#^*p* < 0.05).

### 3.5. Effects of SMT on TNF-*α* Induced Intracellular ROS Production

**Figure 5:**
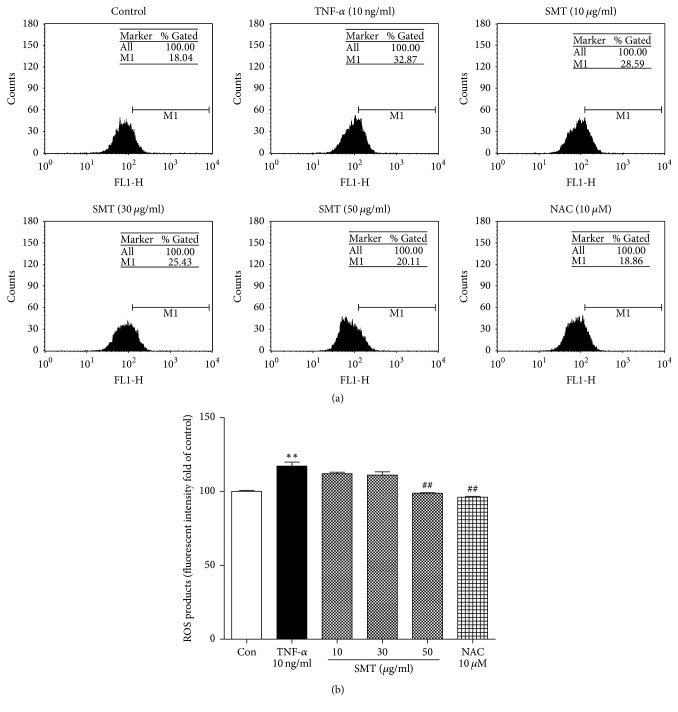
Effect of SMT on TNF-*α* stimulated SMCs ROS production was measured by (a) FACs analysis and (b) microplate reader. Cells were labeled with CM-H_2_DCFDA and treated with TNF-*α* (10 ng/ml) for 24 h in the absence or pretreatment of SMT (10, 30, and 50 *μ*g/ml) for 30 min. N-acetyl-L-cysteine (NAC) was used as positive control. ^*∗∗*^*p* < 0.01 versus untreated control group. ^##^*p* < 0.01 versus TNF-*α*-treated group.

Oxidant stress is a major cause of endothelial dysfunction and vascular inflammation. To measure intracellular ROS production, SMCs were labeled with CM-H_2_DCFDA as described in Materials and methods. As shown in Figure 5, SMCs production of intracellular ROS was increased by TNF-*α* stimulation (^*∗∗*^*p* < 0.01), whereas ROS scavenger N-acetyl-L-cysteine (NAC, 10 *μ*M) inhibited production of intracellular ROS and SMT (10–50 *μ*g/mL) also suppressed production of intracellular ROS (^##^*p* < 0.01), implicating its antioxidant effects.

## 4. Discussion

This study demonstrates that SMT suppressed abnormal increase of SMCs proliferation and migration via regulating cell cycle, ROS production, and MMPs expression against TNF-*α* stimulation. We stimulated SMCs with TNF-*α* to make atherogenic circumstances. Cytokines such as TNF-*α* work as an autocrine and paracrine mediator and are highly expressed in atherosclerotic lesions [16]. Besides, TNF-*α* also leads to production of other inflammatory factors inducing proliferation and migration of SMCs [17]. Proliferation of SMCs was measured with [^3^H]-thymidine incorporation assay in this study, which is used as an index of DNA synthesis. SMT significantly suppressed SMCs [^3^H]-thymidine incorporation against TNF-*α* stimulation in a dose-dependent manner. Thus, detailed antiproliferative mechanism of SMT on TNF-*α* stimulated SMCs cell cycle was investigated.

Phases of the cell cycle are coordinated by CDKs which have little activity in the absence of cyclins. CDKs bind cyclins to form cyclin-CDK complexes and their activities are up to phosphorylation of CDKs and cyclin expression. In quiescent G0 phase, E2F family members exist in inactive form with retinoblastoma protein. However, after mitogenic stimulation, cyclin D1-CDK4 and cyclin E-CDK2 complexes phosphorylate retinoblastoma, dissociating E2F to express other cyclins and CDKs [18]. Cell cycle regulators are key molecules in cancers and atherosclerosis. They induce cell proliferation and share a similar pathogenic pathway [19]. IBRANCE® (Palbociclib) is an FDA-approved drug clinically used in cancer therapy [20]. Working mechanism of Palbociclib is to inhibit CDK4 and CDK6. Rapamycin, an antibiotic produced by* Streptomyces hygroscopicus* [21], is known to inhibit migration of SMCs [22]. Its proposed mechanisms are accumulation of p27^kip1^ and inhibition of CDK activity [23]. In this way, regulating cell cycle of target molecules could be a reasonable pharmacological approach to treat not only cancer but also atherosclerosis. In this study, SMT significantly suppressed the SMCs upregulation of cyclin D1-CDK4 and cyclin E-CDK2 against TNF-*α* stimulation. Furthermore, p21^waf1/cip1^ and p27^kip1^ (CDK inhibitors) expression of SMCs after TNF-*α* stimulation was significantly decreased. However, SMT also showed cell cycle regulative effects by significantly suppressing downregulation of p21^waf1/cip1^ and p27^kip1^ against TNF-*α* stimulation.

Besides, results of migration assay with microscopy showed that SMT suppressed SMCs migration against TNF-*α* stimulation. In addition to cell cycle regulative effects of SMT, underlying mechanism of this phenomenon seems to be resulting from MMP suppressive effects of SMT. SMCs can produce proteolytic enzymes related to the remodeling of MMPs and ECM. In particular, MMP2 and MMP9 belong to representative MMPs. Recent knock-out study also suggests that MMP2 [24] and MMP9 [25] are crucial in development of arterial lesions resulting in atherosclerosis. In ECM remodeling process, new extra matrix components are synthesized and vulnerable lesions are broken down. During the process, activated SMCs abnormally proliferate and migrate into luminal side of blood vessel wall to form thrombus with foam cells that result from endothelial dysfunction [26]. In this study, we performed gelatin zymography and western blot to assay secretion and expression of MMP2 and MMP9. Results of zymography showed that SMT suppressed the SMCs secretion of both MMP2 and MMP9 against TNF-*α* stimulation. In addition, SMT significantly suppressed the protein expression of both MMP2 and MMP9 against TNF-*α* stimulation.

It is suggested that there is a certain relation between oxidative stress and MMPs modulation. Recent studies suggest that MMP-mediated ECM remodeling could be modulated by ROS and one* in vitro* study suggests that oxidative stress may enhance MMP expression and activity [27]. In this study, TNF-*α* stimulation increased the ROS production of SMCs and this might be influence the overall signaling pathways of proliferation and migration. In this regard, SMT suppressed production of intracellular ROS on TNF-*α* stimulated SMCs and it might contribute to cell cycle regulation and MMPs suppression by SMT.

Our previous study demonstrated that SMT attenuated inflammation of vascular endothelial cells against TNF-*α* stimulation via inhibiting activation of nuclear factor-*κ*B and inducing heme oxygenase-1 [15]. In condition of retained vascular dysfunction, vascular inflammation is initiated and results in activation of SMCs. Activated SMCs lose control of cell cycle regulation and migrate into intima resulting in formation of atheroma [8, 28]. Likewise, since this succession of pathogenesis is inseparable, we conducted further study with SMCs to broaden the understanding about vascular protective effects of SMT. To relate our previous and present studies together, SMT not only has protective effects against chronic inflammation on vascular endothelium in early stage but also is expected to suppress abnormal migration of vascular smooth muscle cells and atheroma formation resulting in atherosclerosis.

To summarize, SMT attenuated abnormal migration of vascular smooth muscle cells via regulating cell cycle and suppressing MMPs expression and reactive oxygen species production. We suggest that SMT, a traditionally used herbal formula consisting of four herbs, might act as a vascular protective drug.

## Abbreviations

SMT: Samul-Tang
HUVECs: Human umbilical vein endothelial cells
TNF-*α*: Tumor necrosis factor-alpha
ROS: Reactive oxygen species.

## References

[1] Alexander M. R., Owens G. K. (2012). Epigenetic control of smooth muscle cell differentiation and phenotypic switching in vascular development and disease. *Annual Review of Physiology*.

[2] Ross R. (1995). Cell biology of atherosclerosis. *Annual Review of Physiology*.

[3] Bertoli C., Skotheim J. M., de Bruin R. A. M. (2013). Control of cell cycle transcription during G1 and S phases. *Nature Reviews Molecular Cell Biology*.

[4] Donjerkovic D., Scott D. W. (2000). Regulation of the G1 phase of the mammalian cell cycle. *Cell Research*.

[5] Agewall S. (2006). Matrix metalloproteinases and cardiovascular disease. *European Heart Journal*.

[6] Newby A. C., Zaltsman A. B. (2000). Molecular mechanisms in intimal hyperplasia. *The Journal of Pathology*.

[7] Henney A. M., Wakeley P. R., Davies M. J. (1991). Localization of stromelysin gene expression in atherosclerotic plaques by in situ hybridization. *Proceedings of the National Acadamy of Sciences of the United States of America*.

[8] Collins T., Read M. A., Neish A. S., Whitley M. Z., Thanos D., Maniatis T. (1995). Transcriptional regulation of endothelial cell adhesion molecules: NF-kappa B and cytokine-inducible enhancers.. *The FASEB Journal*.

[9] Yeh L. L. L., Liu J.-Y., Liu Y.-S., Lin K.-S., Tsai T.-F., Wang L.-H. (2009). Anemia-related hemogram, uterine artery pulsatility index, and blood pressure for the effects of four-agents-decoction (Si Wu Tang) in the treatment of primary dysmenorrhea. *The Journal of Alternative and Complementary Medicine*.

[10] Yeh L. L. L., Liu J.-Y., Lin K.-S. (2007). A randomised placebo-controlled trial of a traditional Chinese herbal formula in the treatment of primary dysmenorrhoea. *PLoS ONE*.

[11] Cheng J.-F., Lu Z.-Y. J., Su Y.-C., Chiang L.-C., Wang R.-Y. (2008). A traditional Chinese herbal medicine used to treat dysmenorrhoea among Taiwanese women. *Journal of Clinical Nursing*.

[12] Lee H.-W., Kim H., Ryuk J. A., Kil K.-J., Ko B. S. (2014). Hemopoietic effect of extracts from constituent herbal medicines of Samul-Tang on phenylhydrazine-induced hemolytic anemia in rats. *International Journal of Clinical and Experimental Pathology*.

[13] Dai Y., But P. P.-H., Chan Y.-P., Matsuda H., Kubo M. (2002). Antipruritic and antiinflammatory effects of aqueous extract from Si-Wu-Tang. *Biological & Pharmaceutical Bulletin*.

[14] Tahara E., Satoh T., Toriizuka K. (1999). Effect of Shimotsu-to (a Kampo medicine, Si-Wu-Tang) and its constituents on triphasic skin reaction in passively sensitized mice. *Journal of Ethnopharmacology*.

[15] Choi E. S., Lee Y. J., Seo C. S. (2016). Vascular Protective Role of Samul-Tang in HUVECs: Involvement of Nrf2/HO-1 and NO. *Evidence-Based Complementary and Alternative Medicine*.

[16] Park K.-Y., Heo T.-H. (2017). Critical role of TNF inhibition in combination therapy for elderly mice with atherosclerosis. *Cardiovascular Therapeutics*.

[17] Tedgui A., Mallat Z. (2006). Cytokines in atherosclerosis: pathogenic and regulatory pathways. *Physiological Reviews*.

[18] Sengupta S., Henry R. W. (2015). Regulation of the retinoblastoma-E2F pathway by the ubiquitin-proteasome system. *Biochimica et Biophysica Acta - Gene Regulatory Mechanisms*.

[19] Israels E. D., Israels L. G. (2000). The cell cycle. *The Oncologist*.

[20] Dhillon S. (2015). Palbociclib: First global approval. *Drugs*.

[21] Geng H., Liu H., Liu J., Wang C., Wen J. (2017). Insights into the metabolic mechanism of rapamycin overproduction in the shikimate-resistant Streptomyces hygroscopicus strain UV-II using comparative metabolomics. *World Journal of Microbiology and Biotechnology*.

[22] Cho J. R., Lee C. Y., Lee J. (2016). MicroRNA-761 inhibits Angiotensin II-induced vascular smooth muscle cell proliferation and migration by targeting mammalian target of rapamycin. *Clinical Hemorheology and Microcirculation*.

[23] Morris-Hanon O., Furmento V. A., Rodríguez-Varela M. S. (2017). The Cell Cycle Inhibitors p21Cip1 and p27Kip1 Control Proliferation but Enhance DNA Damage Resistance of Glioma Stem Cells. *Neoplasia (United States)*.

[24] Kuzuya M., Kanda S., Sasaki T. (2003). Deficiency of gelatinase a suppresses smooth muscle cell invasion and development of experimental intimal hyperplasia. *Circulation*.

[25] Cho A., Reidy M. A. (2002). Matrix metalloproteinase-9 is necessary for the regulation of smooth muscle cell replication and migration after arterial injury. *Circulation Research*.

[26] Dzau V. J., Braun-Dullaeus R. C., Sedding D. G. (2002). Vascular proliferation and atherosclerosis: new perspectives and therapeutic strategies. *Nature Medicine*.

[27] Galis Z. S., Khatri J. J. (2002). Matrix metalloproteinases in vascular remodeling and atherogenesis: the good, the bad, and the ugly. *Circulation Research*.

[28] Bennett M. R., Sinha S., Owens G. K. (2016). Vascular smooth muscle cells in atherosclerosis. *Circulation Research*.

